# Systemic Cellular Activation Mapping of an Extinction-Impaired Animal Model

**DOI:** 10.3389/fncel.2019.00099

**Published:** 2019-03-19

**Authors:** Kwanghoon Park, ChiHye Chung

**Affiliations:** Department of Biological Sciences, Konkuk University, Seoul, South Korea

**Keywords:** fear conditioning, extinction, c-Fos, 129S1, C57BL/6, PTSD, animal model

## Abstract

Fear extinction diminishes conditioned fear responses and impaired fear extinction has been reported to be related to anxiety disorders such as post-traumatic stress disorder (PTSD). We and others have reported that 129S1/SvImJ (129S1) strain of mice showed selective impairments in fear extinction following successful auditory or contextual fear conditioning. To investigate brain regions involved in the impaired fear extinction of 129S1 mice, we systemically analyzed c-Fos expression patterns before and after contextual fear conditioning and extinction. After fear conditioning, 129S1 mice showed significantly increased c-Fos expression in the medial division of the central amygdala (CEm), prelimbic (PL) cortex of the medial prefrontal cortex (mPFC), and dorsal CA3 of the hippocampus, compared to that of control C57BL/6 mice. Following fear extinction, 129S1 mice exhibited significantly more c-Fos-positive cells in the CEm, PL, and paraventricular nucleus of the thalamus (PVT) than did C57BL/6 mice. These results reveal the dynamic circuitry involved in different steps of fear memory formation and extinction, thus providing candidate brain regions to study the etiology and pathophysiology underlying impaired fear extinction.

## Introduction

Fear conditioning is a form of fear learning and it associates conditioned stimulus (CS) such as a neutral tone (auditory fear conditioning) or a neutral context (contextual fear conditioning) with an aversive stimulus such as an electrical foot shock (unconditioned stimulus, US). After fear conditioning, the subjects exhibit conditioned fear responses such as freezing behavior to the CS which they were not afraid before conditioning (Pavlov, 1927; Maren et al., 2013). The pairing between CS and US could be unassociated by fear extinction when the CS is presented repeatedly in the absence of the US and after fear extinction, the subjects show diminished fear responses to the CS (Pavlov, 1927; Myers and Davis, 2007; Maren et al., 2013).

Neural mechanisms underlying fear extinction have been explored because of its clinical relevance in post-traumatic stress disorder (PTSD; Herry et al., 2010). Patients with PTSD have been reported to have trouble in overcoming previously formed fear memories and experience impaired fear extinction (Milad et al., 2008, 2009; American Psychiatric Association, 2013; Garfinkel et al., 2014). In this context, the 129S1/SvImJ (129S1) strain of mice can serve as a good animal model to study the etiology of impaired fear extinction. Unlike common strains of mice, 129S1 mice are reported to have difficulty in fear extinction after auditory or contextual fear conditioning (Hefner et al., 2008; Camp et al., 2009; Wille et al., 2015).

Thus far, several brain areas have been implicated in each step of fear memory formation and extinction; these include the lateral amygdala (LA), basal amygdala (BA), lateral division of the central amygdala (CEl), medial division of the central amygdala (CEm), prelimbic (PL) and infralimbic (IL) cortex of the medial prefrontal cortex (mPFC), hippocampus, and paraventricular nucleus of the thalamus (PVT; Milad and Quirk, 2002; Lee and Kesner, 2004; Likhtik et al., 2005; Vidal-Gonzalez et al., 2006; Herry et al., 2008; Burgos-Robles et al., 2009; Hunsaker et al., 2009; Haubensak et al., 2010; Arruda-Carvalho and Clem, 2015; Bukalo et al., 2015; Do-Monte et al., 2015a,b; Kim et al., 2015). Among these, the LA, BA, CEl, CEm, PL, hippocampus, and PVT are involved in fear conditioning, whereas the LA, BA, CEl, hippocampus, and IL mediate fear extinction. Here, we aimed to reveal the neural substrates mediating impaired fear extinction at the cellular level so to provide a systemic framework for future PTSD research. By employing the 129S1 mouse, we monitored changes in brain-wide cellular activation at different stages of contextual fear conditioning and extinction.

## Materials and Methods

### Subjects

Eight to ten-week old male 129S1/SvImJ (129S1) and C57BL/6N (C57BL/6) mice were used for all experiments. 129S1 mice were obtained from the Jackson Laboratory (Bar Harbor, ME, USA) and bred under a pool trio mating system as suggested (Jackson Laboratory). C57BL/6 mice were obtained from Orient Bio (Gapyeong, South Korea) on demand. The mice were group-housed (four mice per cage) under standard laboratory conditions in a humidity—(45%) and temperature—(23 ± 1°C) controlled vivarium on a 12-h light-dark cycle (lights on at 7 a.m.). Mice had free access to food and water. Behavioral experiments were conducted in the daytime starting at around 11 a.m. This study was carried out in accordance with the recommendations of the Guide for the Care and Use of Laboratory Animals of the National Institutes of Health. All experimental procedures related to animals were approved by the Institutional Animal Care and Use Committee of Konkuk University, Seoul, South Korea.

### Auditory Fear Conditioning and Extinction

For auditory fear conditioning, after 5-min of habituation, mice received three pairings of a tone (conditioned stimulus, CS, 75 dB, 10,000 Hz, 30 s) and a foot shock (US, 0.6 mA, 2 s) that was finished with the tone with 20–40 s of inter-stimulus intervals (ISIs) in the conditioning context (H10-11M-TC, Coulbourn Instruments, Whitehall, PA, USA). After the last shock, mice stayed in the context for another 30 s and were then moved back to their home cages (HCs). Fear extinction was performed in the extinction context (acrylic hexagonal prism with an apothem of 11 cm and height of 29 cm) 24 h later. Mice were acclimated for 2 min to the context and were then exposed to a shock-free tone (CS, 75 dB, 10,000 Hz, 30 s) 30 times with 30-s ISIs. After the last tone presentation, mice stayed in the extinction context for 30 s and were then returned to their HCs. The same extinction protocol was repeated 24 h later to complete a 2-day extinction protocol. Freezing behavior was analyzed every 2 s manually by assessing movements excluding respiration (*N* = 4 for each strain).

### Contextual Fear Conditioning and Extinction

For contextual fear conditioning, after 5-min of habituation, mice were exposed to a foot shock (US, 0.6 mA, 2 s) three times with 30-s ISIs in the conditioning context (H10-11M-TC, Coulbourn Instruments). After the last shock, mice stayed in the context for an additional 30 s and were then returned to their HCs. Fear extinction was conducted 24 h later in the same conditioning context for two consecutive days. Mice were placed in the context without shocks for 30 min. The protocol was repeated the next day. Freezing behavior was assessed every 2 s manually (*N* = 6 for each strain).

### Immunohistochemistry

For c-Fos immunoreactivity experiments, there were five groups: HC (*N* = 3 for each strain), context exposure only (*N* = 3 for each strain), fear conditioning (*N* = 5 for each strain), fear extinction 1 (*N* = 5 for each strain), and 2 (*N* = 4 for C57BL/6 mice, *N* = 5 for 129S1 mice). Animals were deeply anesthetized with isoflurane and transcardially perfused with 0.01 M phosphate-buffered saline (PBS), followed by cold 4% paraformaldehyde (PFA) dissolved in 0.01 M PBS 45 min after the end of each condition with the exception of animals of HC group. Brains were then removed and post-fixed with 4% PFA for 24 h. The brains then were transferred to 30% sucrose solution until they sank (i.e., 4% PFA was completely replaced with 30% sucrose). The sunk brains were cryosectioned as 50-μm slices using a microtome and were stored in cryoprotectant at −20°C. Diaminobenzidine (DAB) immunohistochemistry for detecting c-Fos-positive cells was conducted as reported previously with anti c-Fos antibody (sc-52, 1:1,000, Santa Cruz Biotechnology), biotinylated anti-rabbit IgG (BA1000, 1:1,000, Vector Laboratories), ExtrAvidin-peroxidase conjugate (E2886, 1:1,000, Sigma-Aldrich), DAB peroxidase substrate kit (Vector Laboratories), and permount reagent (SP15-500, Fisher Scientific) (Park et al., 2017b). Images were obtained using a microscope (BX51, Olympus) with an attached digital microscope camera (DP72, Olympus). On the images, each brain area was outlined being guided by the Allen mouse brain atlas. With ImageJ software, we measured the size of each brain area and counted c-Fos positive cells in the area manually. It enabled us to calculate how many c-Fos positive cells were there in an area of 1 mm^2^. The number of c-Fos positive cells from two to six brain slices were averaged per mouse.

### Statistical Analysis

The data are presented as mean ± SEM. Statistical tests were performed with GraphPad Prism 8.0.2 and Excel. One-way analysis of variance (ANOVA) was used to assess the performance of each strain in fear conditioning and extinction. Multiple *t*-tests were used to analyze data on c-Fos-expressing neurons between strains for each behavioral manipulation. Strain × condition interactions were analyzed by two-way ANOVA. Comparisons within each strain were analyzed with Dunnett’s multiple comparisons test following two-way ANOVA. *P*-values less than 0.05 were considered statistically significant.

## Results

### 129S1 Mice Exhibited Successful Auditory Fear Conditioning but Impaired Extinction

To validate whether 129S1 mice show intact auditory fear conditioning but impaired fear extinction as reported previously, prior to using 129S1 mice as an animal model to study impaired fear extinction, we measured freezing behavior of 129S1 mice during auditory fear conditioning and extinction (Supplementary Figure S1A), along with C57BL/6 mice, a strain of mice that is widely used for fear conditioning and extinction (Hefner et al., 2008; Camp et al., 2009). There was a significant effect of conditioning trial on freezing acquisition for both 129S1 and C57BL/6 mice (*F*_(2,9)_ = 4.26, *p* < 0.001), suggesting that both strains of mice showed increased levels of freezing behavior across conditioning trials. There was no significant interaction between strain and conditioning trial (*F*_(2,18)_ = 3.55, *p* > 0.3). Extinction was conducted 24 h later for two consecutive days; 129S1 mice had an impairment in fear extinction (*F*_(11,36)_ = 2.07, *p* > 0.7), while C57BL/6 mice showed intact extinction (*F*_(11,36)_ = 2.07, *p* < 0.001). There was a significant interaction between strain and extinction trial/block for freezing behavior (*F*_(11,72)_ = 1.92, *p* < 0.001), indicating that 129S1 mice had impaired auditory fear extinction compared to that of C57BL/6 mice (Supplementary Figure S1B).

### 129S1 Mice Exhibited Normal Contextual Fear Conditioning but Impaired Extinction

Next, we assessed the levels of freezing behavior during contextual fear conditioning and extinction (Supplementary Figure S1C). There was a significant effect of conditioning trial on freezing acquisition for both strains of mice (*F*_(3,20)_ = 3.10, *p* < 0.001). There was no significant interaction between strain and conditioning trial (*F*_(3,40)_ = 2.84, *p* > 0.7). Fear extinction was conducted 24 h later for two consecutive days. 129S1 mice showed impaired extinction (*F*_(3,20)_ = 3.10, *p* > 0.6), while C57BL/6 mice showed no deficits in fear extinction (*F*_(3,20)_ = 3.10, *p* < 0.01). There was a significant interaction between strain and extinction trial/block for the level of freezing (*F*_(3,40)_ = 2.84, *p* < 0.01), implying selective impairment of contextual fear extinction in 129S1 mice compared to C57BL/6 mice (Supplementary Figure S1D). Taken together, these observations confirm that both 129S1 and C57BL/6 mice can be well-trained in contextual fear conditioning, while 129S1 mice have impairments in extinguishing previously formed contextual fear memories.

### Systematic Analysis of Contextual Fear Conditioning and Extinction-Related c-Fos Expression in 129S1 and C57BL/6

**Figure 1:**
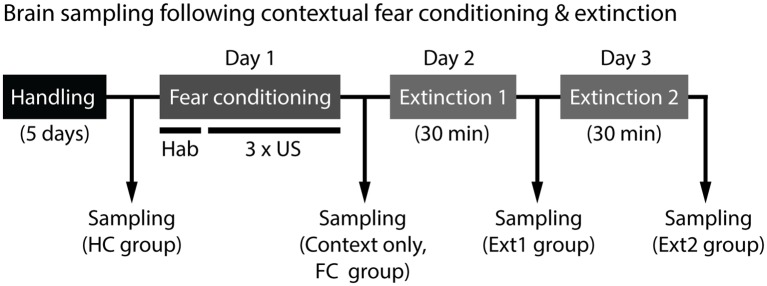
Experimental scheme for brain sampling to analyze c-Fos expression. Brains were collected from mice 45 min after each condition except for home cage (HC) group, where mice were taken directly from their HCs. Contextual fear conditioning consisted of 5-min of habituation (hab) and three unconditioned stimulus (US) exposures (foot shock, 0.6 mA, 2 s) in the conditioning context. Fear extinction was performed 24 h later for 2 days. Mice were placed in the same conditioning context without shocks for 30 min on two consecutive days.

To investigate brain areas involved in disrupted fear extinction of 129S1 mice, we measured and compared expression of an immediate early gene, c-Fos, in the brains of 129S1 and C57BL/6 mice under five different conditions. Two control conditions consisted of mice taken directly from their HCs and mice exposed to the conditioning context without shocks. The other three conditions were mice after fear conditioning, extinction 1, and extinction 2. The brain samples were collected 45 min after each condition except for the HC group (Figure 1). For 129S1 mice, since c-Fos expression after extinction of auditory fear conditioning has been reported previously (Hefner et al., 2008; Whittle et al., 2010), we focused on c-Fos expression following contextual fear conditioning and extinction. c-Fos quantification was performed in the mPFC, LA, BA, CEl, CEm, dorsal hippocampus, ventral hippocampus, PVT, and lateral habenula (LHb).

### CEm of the Amygdala Activation After Fear Extinction Was Higher in 129S1 Mice Than in C57BL/6 Mice

**Figure 2:**
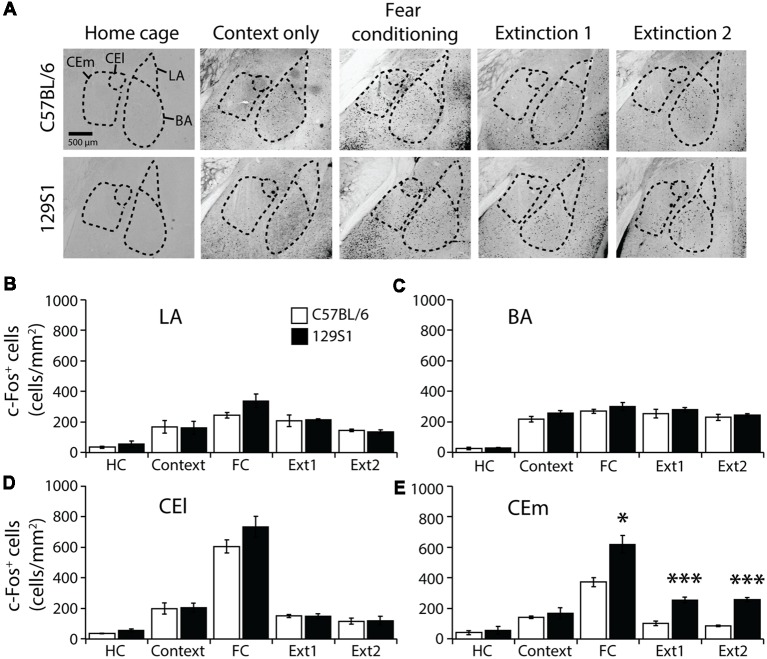
c-Fos expression following contextual fear conditioning and extinction in the amygdala.** (A)** Representative images of c-Fos-positive cells in the lateral amygdala (LA), basal amygdala (BA), lateral division of the central amygdala (CEl), and medial division of the central amygdala (CEm). **(B)** In the LA, 129S1 mice presented similar expression levels of c-Fos in all conditions to C57BL/6 mice. **(C)** In the BA, 129S1 and C57B/6 mice showed comparable numbers of c-Fos-positive cells both at the basal state and after fear conditioning and extinction. **(D)** In the CEl, both 129S1 and C57BL/6 mice exhibited comparable expression of c-Fos in all conditions. **(E)** In the CEm, 129S1 mice expressed significantly more c-Fos positive cells than did C57BL/6 mice after fear conditioning (*p* < 0.05), extinction 1 (*p* < 0.001), and extinction 2 (*p* < 0.001). *p < 0.05, ***p < 0.001.

The amygdala (Bregma −1.26 to −1.56 mm) was divided into four subregions, LA, BA, CEl, and CEm based on the Allen mouse brain atlas, and c-Fos positive cells were quantified (Table 1). In the LA, there was no significant strain × group interaction for c-Fos expression (*F*_(4,31)_ = 1.222, *p* > 0.3). C57BL/6 mice exhibited increased expression of c-Fos after context exposure only (*p* < 0.05), fear conditioning (*p* < 0.001), and fear extinction 1 (*p* < 0.01), compared to the HC group of C57BL/6 mice. Other groups of 129S1 mice also demonstrated elevated expression of c-Fos following fear conditioning (*p* < 0.001), and fear extinction 1 (*p* < 0.01), compared to the HC group. C57BL/6 and 129S1 mice showed similar levels of c-Fos-positive cells throughout the conditions (Figures 2A,B). In the BA, no significant strain × group interaction for c-Fos expression was analyzed (*F*_(4,31)_ = 0.209, *p* > 0.9). All other groups of C57BL/6 mice, including context exposure only (*p* < 0.001), fear conditioning (*p* < 0.001), fear extinction 1 (*p* < 0.001), and fear extinction 2 (*p* < 0.001), presented significantly more c-Fos expression compared to the HC group of C57BL/6 mice. Following context exposure only (*p* < 0.001), fear conditioning (*p* < 0.001), fear extinction 1 (*p* < 0.001), and fear extinction 2 (*p* < 0.001), 129S1 mice exhibited higher levels of c-Fos expression than that of the HC group of the same strain. However, there were no differences between the strains in all the conditions (Figures 2A,C). In the CEl, we found that there was no strain × group interaction for c-Fos expression (*F*_(4,31)_ = 1.256, *p* > 0.3). C57BL/6 mice showed more c-Fos positive cells after context exposure only (*p* < 0.05), and both C57BL/6 and 129S1 mice (*p* < 0.001) presented increased c-Fos expression following fear conditioning compared to each HC group; however, there was no difference between strains (Figures 2A,D). In the CEm, there was a significant strain ± group interaction for c-Fos expression (*F*_(4,31)_ = 5.074, *p* < 0.01). For both C57BL/6 and 129S1 mice, compared to each HC group, there were significantly more c-Fos positive cells following fear conditioning (*p* < 0.001). Increased c-Fos expression was only reduced in C57BL/6 mice following fear extinction 1 (*p* > 0.4) and 2 (*p* > 0.6) to levels comparable with those of the HC group, while 129S1 mice showed consistently increased c-Fos expression even after extinction 1 (*p* < 0.001) and extinction 2 (*p* < 0.001). Significant differences in c-Fos expression between C57BL/6 and 129S1 mice following fear conditioning (*p* < 0.05), fear extinction 1 (*p* < 0.001) and 2 (*p* < 0.001) were observed (Figures 2A,E).

**Table 1:** c-Fos expression of C57BL/6 and 129S1 mice before and after contextual fear conditioning and extinction.

	C57BL/6					129S1				
	HC	Context	FC	Ext1	Ext2	HC	Context	FC	Ext1	Ext2
LA	34 ± 8	168 ± 42	243 ± 19	206 ± 37	144 ± 9	56 ± 20	162 ± 43	337 ± 45	215 ± 7	136 ± 11
BA	26 ± 9	218 ± 17	269 ± 12	255 ± 28	229 ± 20	28 ± 4	255 ± 18	299 ± 27	279 ± 15	243 ± 12
CEl	36 ± 1	199 ± 36	605 ± 43	150 ± 11	116 ± 19	56 ± 9	203 ± 30	733 ± 70	149 ± 16	120 ± 29
CEm	41 ± 13	140 ± 8	372 ± 29	101 ± 15	86 ± 4	57 ± 24	168 ± 37	618 ± 58*	255 ± 19***	257 ± 12***
PL	24 ± 5	237 ± 14	451 ± 19	345 ± 60	225 ± 16	30 ± 10	306 ± 30	731 ± 83*	603 ± 27*	512 ± 59*
IL	22 ± 5	334 ± 7	455 ± 37	504 ± 54	300 ± 51	29 ± 11	388 ± 49	491 ± 27	436 ± 24	335 ± 44
dCA1	60 ± 8	142 ± 12	237 ± 30	195 ± 10	155 ± 15	65 ± 11	125 ± 8	264 ± 47	171 ± 27	146 ± 10
dCA3	66 ± 2	142 ± 10	174 ± 4	162 ± 19	163 ± 21	73 ± 5	161 ± 20	240 ± 6***	229 ± 22	194 ± 9
dDG	71 ± 3	169 ± 18	156 ± 9	153 ± 16	145 ± 31	79 ± 5	176 ± 11	192 ± 20	171 ± 18	179 ± 18
vCA1	70 ± 1	136 ± 11	170 ± 15	169 ± 14	139 ± 7	66 ± 1	132 ± 10	174 ± 8	157 ± 8	140 ± 8
vCA3	86 ± 2	123 ± 13	167 ± 6	162 ± 10	143 ± 3	66 ± 7	148 ± 30	195 ± 20	171 ± 10	160 ± 5
vDG	62 ± 5	103 ± 8	130 ± 10	118 ± 11	78 ± 1	71 ± 1	100 ± 8	143 ± 8	141 ± 5	95 ± 11
PVT	60 ± 11	353 ± 65	483 ± 61	480 ± 91	229 ± 35	68 ± 18	433 ± 47	781 ± 130	721 ± 81	597 ± 81*
LHb	58 ± 8	267 ± 41	309 ± 38	302 ± 30	263 ± 21	62 ± 7	265 ± 42	329 ± 43	345 ± 42	309 ± 28

The c-Fos expression data are presented as mean ± SEM for c-Fos^+^ cells/mm^2^. *p < 0.05, ***p < 0.001, between C57BL/6 and 129S1 mice.

### PL of the mPFC Was Activated in 129S1 Mice but Not C57BL/6 Mice After Fear Extinction

**Figure 3:**
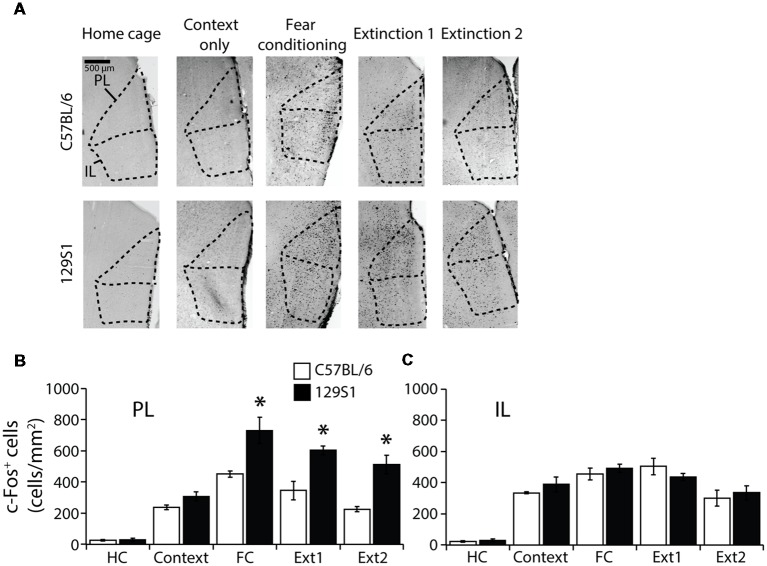
c-Fos expression following contextual fear conditioning and extinction in the medial prefrontal cortex (mPFC).** (A)** Representative images of c-Fos positive cells in the prelimbic (PL) cortex and infralimbic (IL) cortex of the mPFC. **(B)** In the PL, 129S1 mice showed more c-Fos positive cells than did C57BL/6 mice following fear conditioning (*p* < 0.05), extinction 1 (*p* < 0.05), and extinction 2 (*p* < 0.05). **(C)** In the IL, 129S1 and C57BL/6 mice expressed comparable c-Fos positive cells in all conditions. *p < 0.05.

The PL and IL of the mPFC (Bregma +1.55 to +1.65 mm) were analyzed to measure c-Fos expression (Table 1). In the PL, we observed a significant strain × group interaction for c-Fos expression (*F*_(4,31)_ = 3.040, *p* < 0.05). C57BL/6 mice showed significantly increased c-Fos expression compared to HC group of the same strain following context exposure only (*p* < 0.05), fear conditioning (*p* < 0.001), extinction 1 (*p* < 0.001), and extinction 2 (*p* < 0.05). 129S1 mice had more expression of c-Fos after context exposure only (*p* < 0.01), fear conditioning (*p* < 0.001), extinction 1 (*p* < 0.001), and extinction 2 (*p* < 0.001) than that of 129S1 mice taken directly from the HC. 129S1 mice showed significantly more c-Fos expression after fear conditioning (*p* < 0.05), extinction 1 (*p* < 0.05), and extinction 2 (*p* < 0.05) than that of C57BL/6 mice (Figures 3A,B). In the IL, no significant strain × group interaction for c-Fos expression was found (*F*_(4,31)_ = 0.834, *p* > 0.5). Both C57BL/6 and 129S1 mice exhibited significantly increased c-Fos expression compared to the same-strain HC group following context exposure only (*p* < 0.001), fear conditioning (*p* < 0.001), extinction 1 (*p* < 0.001), and extinction 2 (*p* < 0.001). No differences on c-Fos expression were observed between the strains (Figures 3A,C).

### Dorsal Hippocampal CA3 Was Consistently Activated in 129S1 Mice After Fear Conditioning

**Figure 4:**
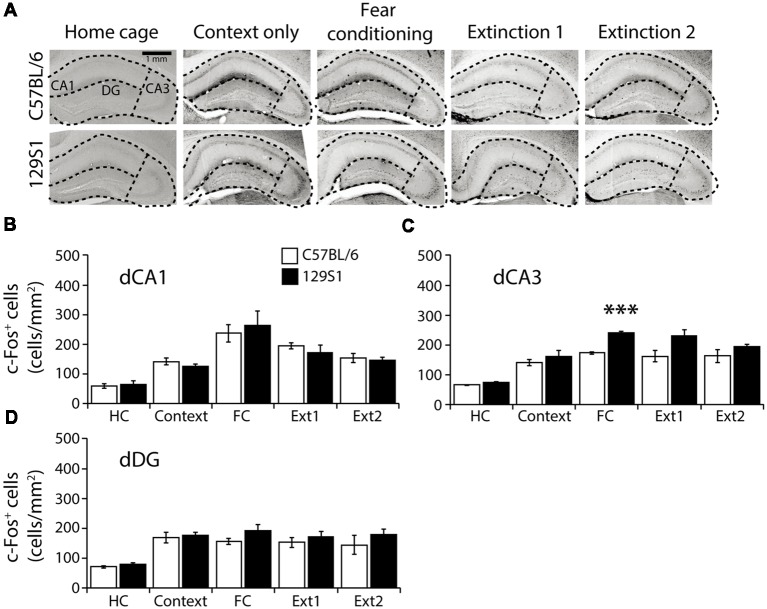
c-Fos expression following fear conditioning and extinction in the dorsal hippocampus.** (A)** Representative images of c-Fos positive cells in the dorsal CA1, CA3, and dentate gyrus (DG) of the hippocampus. **(B)** In the dorsal CA1, C57BL/6 and 129S1 mice showed comparable expression of c-Fos. **(C)** In the dorsal CA3, compared to C57BL/6 mice, 129S1 mice showed significantly more c-Fos-positive cells after fear conditioning (*p* < 0.001). **(D)** In the dorsal DG, no differences were observed in c-Fos expression of C57BL/6 and 129S1 mice. ***p < 0.001.

In the dorsal CA1 of the hippocampus (Bregma −1.86 to −1.96 mm; Table 1), there was no significant strain × group interaction for c-Fos expression (*F*_(4,31)_ = 0.355, *p* > 0.8). C57BL/6 and 129S1 mice showed similar levels of c-Fos expression in all five conditions. Compared to each HC group, both C57BL/6 and 129S1 mice exhibited increased c-Fos expression following fear conditioning (*p* < 0.001) and extinction 1 (*p* < 0.01 for C57BL/6 mice, *p* < 0.05 for 129S1 mice; Figures 4A,B). In the dorsal CA3, no significant strain × group interaction for c-Fos expression was observed (*F*_(4,31)_ = 1.611, *p* > 0.1). Both C57BL/6 and 129S1 mice showed more c-Fos expression following context exposure only (*p* < 0.05 for C57BL/6 mice, *p* < 0.01 for 129S1 mice), fear conditioning (*p* < 0.001), extinction 1 (*p* < 0.001), and extinction 2 (*p* < 0.001) compared to that of the HC group. Between C57BL/6 and 129S1 mice, 129S1 mice showed more c-Fos expression following fear conditioning (*p* < 0.001; Figures 4A,C). In the dorsal dentate gyrus (DG), we found no significant strain × group interaction for c-Fos expression (*F*_(4,31)_ = 0.271, *p* > 0.8). Compared to HC groups of each strain, both C57BL/6 and 129S1 mice expressed more c-Fos following context exposure only (*p* < 0.05), fear conditioning (*p* < 0.05 for C57BL/6 mice, *p* < 0.01 for 129S1 mice), extinction 1 (*p* < 0.05 for C57BL/6 mice, *p* < 0.01 for 129S1 mice) and extinction 2 (*p* < 0.05 for C57BL/6 mice, *p* < 0.01 for 129S1 mice). No differences on c-Fos expression were detected between C57BL/6 and 129S1 mice (Figures 4A,D).

### Ventral Hippocampus of 129S1 Mice Showed Comparable c-Fos Expression to C57BL/6 Mice

**Figure 5:**
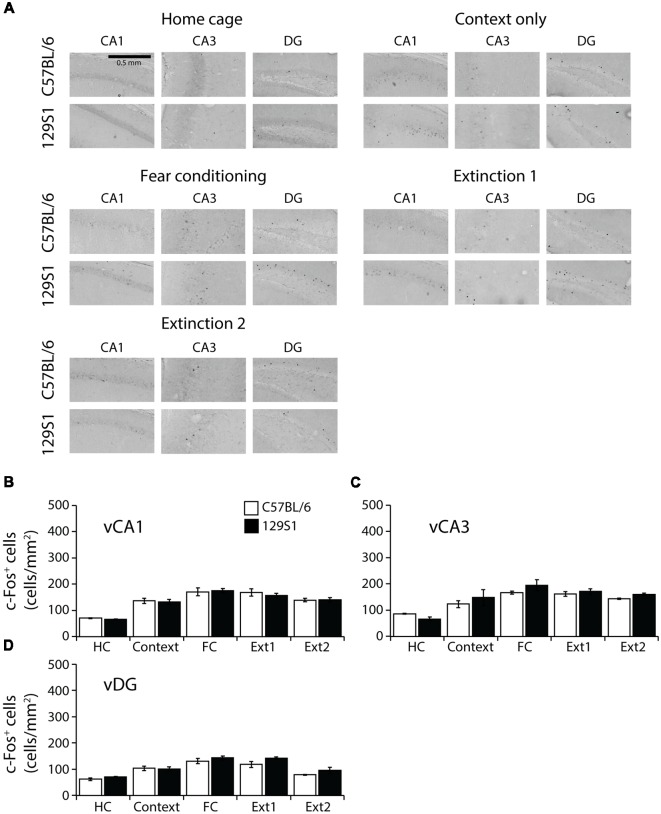
c-Fos expression following fear conditioning and extinction in the ventral hippocampus.** (A)** Representative images of c-Fos positive cells in the ventral CA1, CA3, and DG of the hippocampus. **(B)** In the ventral CA1, C57BL/6 and 129S1 mice presented similar levels of c-Fos. **(C)** In the ventral CA3, no differences in the number of c-Fos positive cells between C57BL/6 and 129S1 mice were found. **(D)** In the ventral DG, C57BL/6 and 129S1 mice showed comparable c-Fos expression.

In the ventral CA1 of the hippocampus (Bregma −2.78 to −2.88 mm; Table 1), there was no significant strain × group interaction for c-Fos expression (*F*_(4,31)_ = 0.203, *p* > 0.9). Both C57BL/6 and 129S1 mice presented more c-Fos positive cells after context exposure only (*p* < 0.01), fear conditioning (*p* < 0.001), extinction 1 (*p* < 0.001), and extinction 2 (*p* < 0.001), compared to those of each HC group (Figures 5A,B). In the ventral CA3, no significant strain × group interaction for c-Fos expression was analyzed (*F*_(4,31)_ = 0.929, *p* > 0.4). 129S1 mice of context exposure only group expressed more c-Fos positive cells than 129S1 mice of HC group did (*p* < 0.01). Compared to each HC group, both C57BL/6 and 129S1 mice exhibited more c-Fos expression following fear conditioning (*p* < 0.001), extinction 1 (*p* < 0.01 for C57BL/6 mice, *p* < 0.001 for 129S1 mice) and extinction 2 (*p* < 0.05 for C57BL/6 mice, *p* < 0.001 for 129S1 mice; Figures 5A,C). In the ventral DG, there was no significant strain × group interaction for c-Fos expression (*F*_(4,31)_ = 0.542, *p* > 0.7). C57BL/6 mice of context exposure only group showed elevated c-Fos expression, compared to HC group of C57BL/6 (*p* < 0.05). Both C57BL/6 and 129S1 mice presented more c-Fos positive cells following fear conditioning (*p* < 0.001) and extinction 1 (*p* < 0.001), compared to each HC group (Figures 5A,D). There were no differences for c-Fos expression between C57BL/6 and 129S1 mice in ventral CA1, ventral CA3, and ventral DG.

### PVT Activation Remained Higher After Fear Extinction in 129S1 Mice but Not in C57BL/6 Mice

**Figure 6:**
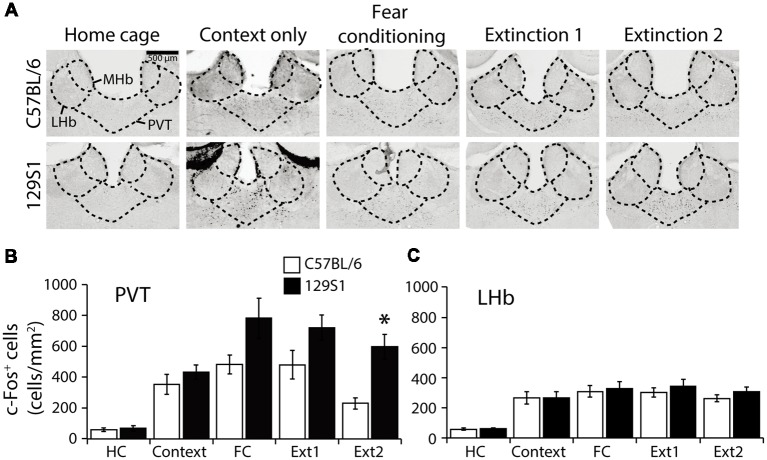
c-Fos expression following fear conditioning and extinction in the paraventricular nucleus of the thalamus (PVT) and lateral habenula (LHb). **(A)** Representative images of c-Fos positive cells in the PVT and LHb. **(B)** In the PVT, 129S1 mice showed more expression of c-Fos after extinction 2 compared to that of C57BL/6 mice (*p* < 0.05). **(C)** In the LHb, both 129S1 and C57BL/6 mice presented similar levels of expression of c-Fos in all conditions. *p < 0.05.

In the PVT (Bregma −1.66 mm; Table 1), we found no significant strain × group interaction for c-Fos expression (*F*_(4,31)_ = 1.388, *p* > 0.2). Compared to the HC group, 129S1 mice exhibited increased expression of c-Fos following context exposure only (*p* < 0.05). Both C57BL/6 and 129S1 mice showed elevated c-Fos positive cells after fear conditioning (*p* < 0.01 for C57BL/6, *p* < 0.001 for 129S1 mice), and extinction 1 (*p* < 0.01 for C57BL/6, *p* < 0.001 for 129S1 mice). C57BL/6 mice exhibited decreased c-Fos expression to basal levels of the HC group after extinction 2 (*p* > 0.4), while 129S1 mice still expressed more c-Fos-positive cells than that of HC group (*p* < 0.001). Between the strains, 129S1 mice showed significantly more c-Fos-positive cells after extinction 2 (*p* < 0.05) than that of C57BL/6 mice (Figures 6A,B). In the LHb (Bregma −1.66 mm; Table 1), there was no significant strain × group interaction for c-Fos expression (*F*_(4,31)_ = 0.174, *p* > 0.9). Both C57BL/6 and 129S1 mice demonstrated increased expression of c-Fos following context exposure only (*p* < 0.01), fear conditioning (*p* < 0.001), extinction 1 (*p* < 0.001), and extinction 2 (*p* < 0.01 for C57BL/6 mice, *p* < 0.001 for 129S1 mice) compared to that of each HC group of the same strain. There were no differences between strains in all the five conditions (Figures 6A,C).

## Discussion

In this study, we investigated the activated brain areas of a fear extinction-impaired animal model, 129S1 mice after contextual fear conditioning and extinction. We examined the brain areas involved in the impaired fear-extinction of 129S1 mice by analyzing an immediate early gene, c-Fos expression throughout the brains and comparing it with that of control C57BL/6 mice.

We observed that 129S1 mice had impaired fear extinction after either auditory or contextual fear conditioning as reported previously (Hefner et al., 2008; Camp et al., 2009; Wille et al., 2015). During fear extinction after auditory or contextual fear conditioning, 129S1 mice exhibited constant freezing behaviors to the CSs; there were significant strain × extinction trial/block interactions in 129S1 mice compared to C57BL/6. These results suggest that 129S1 mice could be a useful animal model to study the etiology underlying impaired extinction in PTSD.

The amygdala is where fear memories are formed and stored, and it is also involved in extinguishing previously formed fear memories (Rogan et al., 1997; LeDoux, 2003; Herry et al., 2008; Ciocchi et al., 2010; Duvarci et al., 2011; Tye et al., 2011; Lee et al., 2013; Krabbe et al., 2018). The amygdalar of PTSD patients are hyperactive not only to traumatic cues, but also to trauma-unrelated affective cues like fearful facial expressions (Liberzon et al., 1999; Rauch et al., 2000; Driessen et al., 2004; Shin et al., 2004; Wright et al., 2007). In this study, we observed significantly different c-Fos expression between C57BL/6 and 129S1 mice in the CEm following contextual fear conditioning and extinction. The observation of hyperactive CEm in 129S1 mice after contextual fear extinction correspond to previously reported data that 129S1 mice express more c-Fos and another immediate early gene, Zif268, in the CEm relative to that of C57BL/6 mice after auditory fear extinction (Hefner et al., 2008; Whittle et al., 2010). As the CEm projects to the periaqueductal gray (PAG) and induces freezing behavior (LeDoux et al., 1988; De Oca et al., 1998; Tye et al., 2011), we conclude that the hyperactive CEm of 129S1 mice after contextual fear extinction may contribute to impaired extinction. Following contextual extinction, we did not observe differences in c-Fos expression in the BA of 129S1 mice compared to C57BL/6 mice, while decreased c-Fos expression after auditory extinction has been reported previously (Hefner et al., 2008; Whittle et al., 2010). Differences between contextual and auditory fear conditioning may underlie this discordance; further, the BA may not be critical for contextual fear memory formation. In the present study, the context exposure only and fear conditioning groups did not show differences in c-Fos expression in the BA for both C57BL/6 and 129S1 mice, which is consistent with a previous study reporting that context exposure only and contextual fear conditioning produce comparable Fos expression in the BA of C57BL/6 mice (Milanovic et al., 1998). It has also been reported that the BA is not critical for cognitive memory of contextual fear conditioning (Vazdarjanova and McGaugh, 1998). Taken together, increased activity of the CEm in 129S1 mice after contextual fear extinction might be involved in impaired contextual fear extinction.

The PL and IL of the mPFC are involved in fear conditioning and extinction, respectively (Milad and Quirk, 2002; Vertes, 2004; Likhtik et al., 2005; Vidal-Gonzalez et al., 2006; Herry et al., 2008; Burgos-Robles et al., 2009; Bukalo et al., 2015; Do-Monte et al., 2015a). We observed that activity in the PL of the mPFC remained higher after contextual fear conditioning and extinction in 129S1 mice, compared to that of C57BL/6 mice. The results following extinction are consistent with previously reported data of Zif268 expression in 129S1 mice after auditory fear extinction (Hefner et al., 2008). It has also been reported that impaired extinction in 129S1 mice was associated with elevated PL single-unit firing (Fitzgerald et al., 2014). As the PL is involved in fear expression rather than fear memory formation, we can conclude that sustained PL activation after contextual fear conditioning and extinction results in sustained fear expression and impaired fear extinction in 129S1 mice (Corcoran and Quirk, 2007; Rozeske et al., 2015). We observed no differences in the IL which is an area related to consolidation of extinction memory between C57BL/6 and 129S1 mice (Vidal-Gonzalez et al., 2006; Do-Monte et al., 2015a). In a previous study, the IL of 129S1 mice expressed less c-Fos after auditory extinction relative to that in C57BL/6 mice. However, another study reported that 129S1 mice had exaggerated IL single-unit firing after extinction (Hefner et al., 2008; Fitzgerald et al., 2014). Therefore, further studies need to clarify the exact activity of the IL in 129S1 mice after extinction.

The hippocampus plays a role in contextual fear memory formation and expression (Phillips and LeDoux, 1992; Sanders et al., 2003). The activity of the dorsal CA3 in the hippocampus remained higher in 129S1 following contextual conditioning. Since the dorsal CA3 is involved in acquisition and retention of contextual fear memory, a hyperactive CA3 in 129S1 mice following fear conditioning could mean stronger fear memories are formed (Lee and Kesner, 2004; Hunsaker et al., 2009). Since the dorsal CA1 and CA3 have been known to be involved in fear extinction as well, the comparable c-Fos expression between C57BL/6 and 129S1 mice in dorsal CA1 and CA3 following fear extinction could be due to the dual roles of the areas participating in both fear conditioning and extinction (Dillon et al., 2008; Ji and Maren, 2008).

Ventral to the hippocampus lies the PVT and LHb. The PVT has recently been proposed to be involved in long-term fear memory storage (Arruda-Carvalho and Clem, 2015; Do-Monte et al., 2015b; Penzo et al., 2015), and the LHb is related to depression-like behaviors (Matsumoto and Hikosaka, 2007, 2009; Li et al., 2011; Park et al., 2017a,b,c). 129S1 mice exhibited higher activity in the PVT after contextual fear extinction. The PVT may have a role in fear memory expression and long-term fear memory storage. As such, a hyperactive PVT in 129S1 mice may contribute to long-lasting fear in 129S1 mice (Arruda-Carvalho and Clem, 2015; Do-Monte et al., 2015b).

In the present study, we explored hyperactive brain areas following contextual fear conditioning and extinction in 129S1 mice, a strain of mice with impaired fear extinction. Our c-Fos expression data contribute to our understanding of the neural substrates underlying impaired fear extinction at the cellular level. This will lay the platform for future fear extinction-related PTSD research and facilitate the search for effective therapeutics for impaired extinction.

## Data Availability

All datasets generated for this study are included in the manuscript and/or the supplementary files.

## Author Contributions

CC conceived this work and designed the experiments. KP performed the experiments and acquired the data. KP and CC analyzed and interpreted the data and prepared the manuscript. Both authors have approved the final version of the manuscript.

## Conflict of Interest Statement

The authors declare that the research was conducted in the absence of any commercial or financial relationships that could be construed as a potential conflict of interest.

## Supplementary Material

FIGURE S1 129S1 mice show impaired extinction following auditory or contextual fear conditioning.** (A)** Experimental scheme for auditory fear conditioning and extinction. **(B)** Both C57BL/6 and 129S1 mice showed normal auditory fear conditioning across conditioning trials. 129S1 mice, however, exhibited impaired fear extinction following auditory fear conditioning, while C57BL/6 mice had normal fear extinction. An extinction block consists of 5 extinction trials. 129S1 mice showed significantly high levels of freezing in the third paring of CS and US in conditioning (*p* < 0.01), extinction block 3 on day 2 (*p* < 0.01), extinction block 1 (*p* < 0.01), and in all the other blocks (*p* < 0.001) on day 3 compared to C57BL/6 mice. **(C)** Experimental scheme for contextual fear conditioning and extinction. **(D)** Normal contextual fear conditioning was seen in both C57BL/6 and 129S1 mice following conditioning trials. 129S1 mice showed impaired fear extinction after contextual fear conditioning, while C57BL/6 mice performed successful fear extinction. 129S1 mice showed higher levels of freezing than C57BL/6 mice in extinction 2 on day 3 (*p* < 0.01 for the first 3 min, *p* < 0.001 for the last 3 min). ***p* < 0.01, ****p* < 0.001.
