# Supplementary figures and images for: Systemic Cellular Activation Mapping of an Extinction-Impaired Animal Model
Source: Front Cell Neurosci. 2019 Mar 19;13:99. doi: 10.3389/fncel.2019.00099 (PMC6433791; doi:10.3389/fncel.2019.00099)

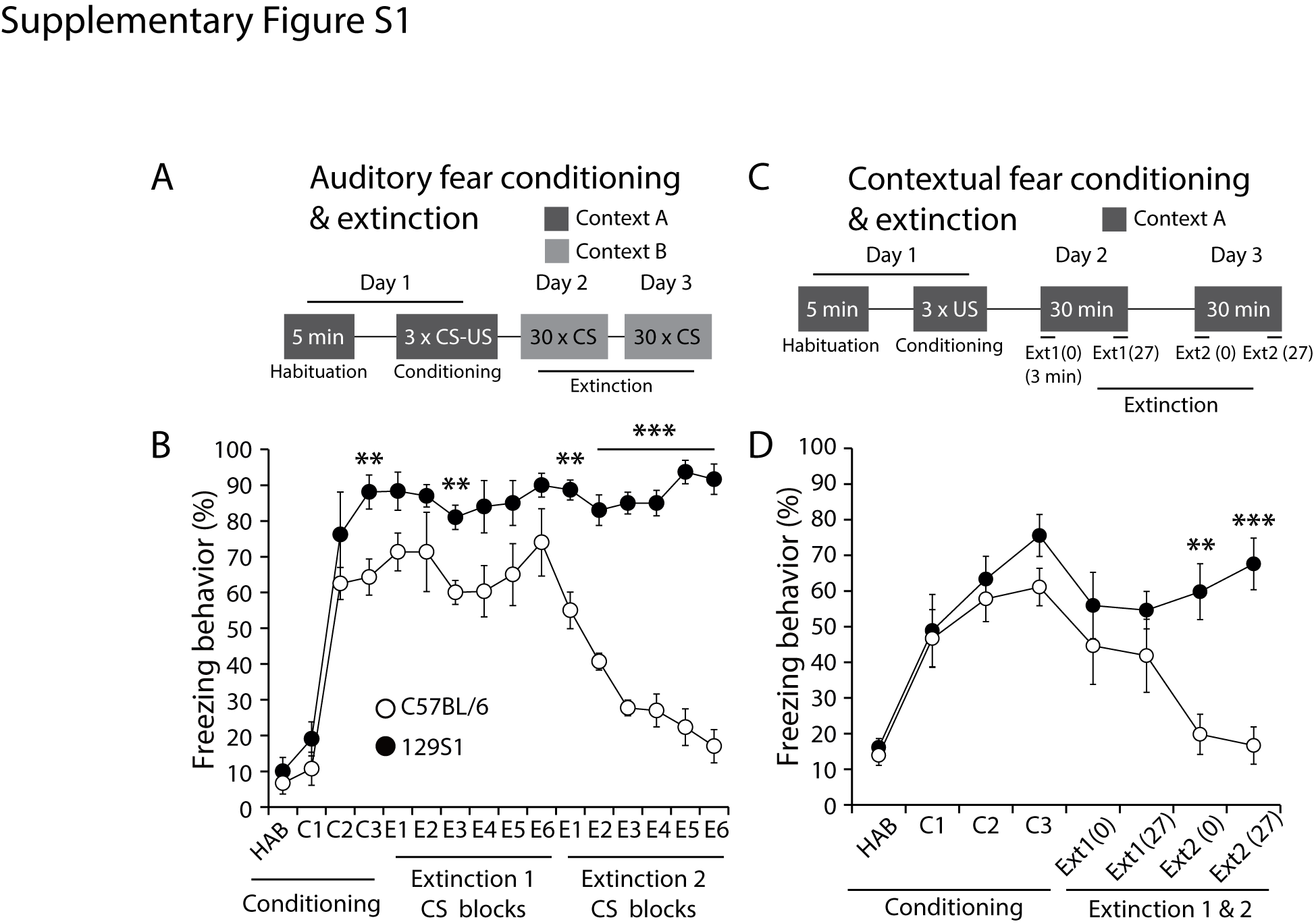

Supplement: FIGURE S1 — 129S1 mice show impaired extinction following auditory or contextual fear conditioning. (A) Experimental scheme for auditory fear conditioning and extinction. (B) Both C57BL/6 and 129S1 mice showed normal auditory fear conditioning across conditioning trials. 129S1 mice, however, exhibited impaired fear extinction following auditory fear conditioning, while C57BL/6 mice had normal fear extinction. An extinction block consists of 5 extinction trials. 129S1 mice showed significantly high levels of freezing in the third paring of CS and US in conditioning (p < 0.01), extinction block 3 on day 2 (p < 0.01), extinction block 1 (p < 0.01), and in all the other blocks (p < 0.001) on day 3 compared to C57BL/6 mice. (C) Experimental scheme for contextual fear conditioning and extinction. (D) Normal contextual fear conditioning was seen in both C57BL/6 and 129S1 mice following conditioning trials. 129S1 mice showed impaired fear extinction after contextual fear conditioning, while C57BL/6 mice performed successful fear extinction. 129S1 mice showed higher levels of freezing than C57BL/6 mice in extinction 2 on day 3 (p < 0.01 for the first 3 min, p < 0.001 for the last 3 min). **p < 0.01, ***p < 0.001. [file Image_1.TIF]
